# Gender specific eRNA TBX5-AS1 as the immunological biomarker for male patients with lung squamous cell carcinoma in pan-cancer screening

**DOI:** 10.7717/peerj.12536

**Published:** 2021-11-25

**Authors:** Tao Yan, Kai Wang, Qidi Zhao, Junjie Zhuang, Hongchang Shen, Guoyuan Ma, Lei Cong, Jiajun Du

**Affiliations:** 1 Institute of Oncology, Shandong Provincial Hospital, Cheeloo College of Medicine, Shandong University, Jinan, China; 2 Department of Healthcare Respiratory Medicine, Shandong Provincial Hospital, Cheeloo College of Medicine, Shandong University, Jinan, China; 3 Institute of Oncology, Shandong Provincial Hospital affiliated to Shandong First Medicine University, Jinan, China; 4 Department of Oncology, Shandong Provincial Hospital affiliated to Shandong First Medicine University, Jinan, China; 5 Department of Thoracic Surgery, Shandong Provincial Hospital, Cheeloo College of Medicine, Shandong University, Jinan, China; 6 Department of Oncology, Shandong Provincial Hospital, Cheeloo College of Medicine, Shandong University, Jinan, China

**Keywords:** Gender, eRNA, Immunotherapy, Lung squamous cell carcinoma, Pan-cancer analysis

## Abstract

As an innate feature of human beings, gender differences have an influence on various biological phenotypes, yet it does not attract enough attention in genomics studies. The prognosis of multiple carcinomas usually exhibits a favorable ending for female patients, but the neglect of gender differences can cause serious bias in survival analysis. Enhancer RNAs (eRNAs) are mostly downstream of androgens or estrogen. The present study was aimed to screen eRNAs in patients with non-small-cell lung cancer. The findings revealed that eRNA TBX5-AS1 was expressed differently between female and male patients. Meanwhile, its prognostic significance appeared only in male patients with squamous cell carcinoma (SCC) type. The Gene Set Enrichment Analysis proved that the expression level of TBX5-AS1 increased following the activation of the androgen signaling pathway. In pan-cancer analysis, the prognostic prediction based on gender grouping obtained more meaningful results, and the synergy between TBX5-AS1 and its homologous target was more consistent. Furthermore, immunity variations between sexes prompted us to explore the role that TBX5-AS1 played in tumor microenvironment and immunotherapy. The robust evidence proved that male patients with high expression of TBX5-AS1 possessed a malignant immune microenvironment and urgently needed immune checkpoint inhibitor treatment. In conclusion, TBX5-AS1 may be one of the strongest candidates to predict prognosis for male patients with SCC and provide a reference for immunotherapy.

## Introduction

Enhancer RNAs (eRNAs) are a group of long noncoding RNAs (lncRNAs) derived from the enhancer regions (Orom et al., 2010; Mao et al., 2019). With bidirectional transcription, the expression levels of eRNA vary following the original enhancer activity (Andersson et al., 2014; Core et al., 2014). Although eRNAs are known for over a decade, they have not received enough attention in the latest RNA reviews (De Santa et al., 2010; Kim et al., 2010; Mao et al., 2019; Goodall & Wickramasinghe, 2021). Certainly, researches on eRNAs are insufficient, and there are currently several potential mechanisms. eRNA could interact with RNA binding proteins (RBP) and regulate gene transcription. Lee summarized some common mechanism models of eRNA-protein binding: chromatin loop, recruitment of acetylated histone reader/writer, trapping transcription factor and regulation of RNA polymerase II pause-release (Lee, Xiong & Li, 2020). Since enhancer dysfuction is considered as a key mechanism of tumorigenesis, eRNAs also regulate expression and function of oncogene and tumor suppressors. Previous studies revealed that eRNA played a vital role in tumorigenesis and progression of lung cancer (Qin et al., 2020), lymphoma (Park et al., 2020), breast cancer (Zhu et al., 2019), prostate cancer (Hsieh et al., 2014), etc. eRNAs are also involved in the regulation of cancer signaling. eRNAs are cancer- or lineage-specific and may be driven by tissue-specific transcript factors (Zhang et al., 2019). That indicated the potential value of eRNAs on the cancer diagnosis and prognosis.

One of the limitations of research on eRNAs is their instability (Andersson et al., 2014). Hojoong Kwak and his colleagues overcame eRNA instability *via* the nascent RNA sequencing (Kristjansdottir et al., 2020), allowing deeper exploration and broader eRNA studies. Recently, studies have found that sex hormones can regulate cell biological behavior by altering the expression of eRNA (Mao et al., 2019). In estrogen-regulated systems, eRNAs are transcribed uni- or bi-directionally from the estrogen receptor (ER) binding sites and strengthen the specific enhancer–promoter looping based on a cohesion-dependent mechanism (Hah et al., 2013). Moreover, eRNA KLK3e needs to be combined with the androgen receptor and mediator 1 to facilitate KLK3 and KLK2 transcription (Hsieh et al., 2014). All together, recent scientific outcomes suggested that eRNAs might exert essential roles in gender differences.

Research derived from The Cancer Genome Atlas (TCGA) database analysis rarely considered gender as a biological variable (Wilson & Buetow, 2020). The search results on “cancer” and “TCGA” reduced by nearly 80 times when the term “gender” was added (Wilson & Buetow, 2020). However, differences were found between male and female in multiple types of cancers (Cook et al., 2009). A Swedish cohort study proved that male patients were at a higher risk for 34 of 39 cancers and possessed a poorer prognosis for 27 of 39 cancers compared with female patients. The cancer types involved were divided into eight categories: head–neck, upper digestive, lower digestive, respiratory, urinary, skin, central nervous system, and hematological (Radkiewicz et al., 2017; Radkiewicz et al., 2020).

Moreover, gender differences also existed in the immune system, including immune response, autoimmune disorders, and infection response (Jacobson et al., 1997; vom Steeg & Klein, 2016; Wang, Cowley & Liu, 2019; Takahashi & Iwasaki, 2021). The inclusion of “gender” as a research variable still faces many challenges, but researchers should always be alert toward bias in gender differences and explore potential mechanisms (Fu et al., 2020a; Wilson & Buetow, 2020).

In 2019, this retrospective study was performed on 8,668 patients with pulmonary carcinoma who underwent resection at The Shandong Provincial Hospitals since January 1, 1990. The results demonstrated that the proportion of lung adenocarcinoma (LUAD) among female nonsmokers increased dramatically (Huang, Qu & Du, 2019). From our clinical practice, non-small-cell lung cancer (NSCLC), which occupied the top spot among many cancers, was selected to screen for eRNAs (Siegel, Miller & Jemal, 2018). Given the widespread existence of gender differences in various tumors, a pan-cancer analysis for TBX5-AS1 was performed. Additionally, the prognostic significance for male patients with lung squamous cell carcinoma (LUSC) was verified with an additional cohort to predict further the response to immune checkpoint inhibitor (ICI) treatment.

## Materials and Methods

### Data preparation and patient samples

The RNA expression file and related clinical information on carcinomas were downloaded from the TCGA database *via* University of California, Santa Cruz (UCSC) Xena (https://xenabrowser.net/). In total, 34 types of solid cancers and the Genotype-Tissue Expression (GTEx) project were included for supplementing normal samples of human organs. The list of cancer types is provided in the Supplemental Files (Table S1). Besides, 364 patients with NSCLC from GSE37745 and GSE50881 were also involved in the study. GSE37745 and GSE50881 were combined *via* batch analysis. Predicting Specific Tissue Interactions of Genes and Enhancers (PreSTIGE) predicted the information on eRNAs and their potential targets (Corradin et al., 2014; Vučićević et al., 2015).

The validation cohort consisted of 30 male patients with squamous cell carcinoma (SCC) and 64 patients with adenocarcinoma (ADC) from the Department of Thoracic Surgery, Shandong Provincial Hospital. The ethics review committee of Shandong Provincial Hospital approved this study (SWYX: No.2021-260). It was performed based on the Declaration of Helsinki and Good Clinical Practice guidelines, as defined by the International Conference on Harmonization.

### Screening of eRNA with prognostic significance and functional enrichment analysis

The Kaplan–Meier (KM) survival curves were used to pick valuable eRNAs in specific cancer types. Pearson correlation coefficients were used to evaluate the correlation between eRNAs and protein-coding genes (PCGs). PCGs that met the standards were considered lncRNA-related PCGs (*p* < 0.001; |Pearson correlation coefficient| > 0.4). The screened PCGs were used for Gene Ontology (GO) and Kyoto Encyclopedia of Genes and Genomes (KEGG) analyses based on R packages colorspace, stringi, ggplot2, enrichplot, clusterProfiler, and DOSE. The proteomics data were obtained from The Cancer Proteome Atlas (TCPA) database to explore the potential downstream targets.

### Assessment of infiltrating immune cells

A computational framework CIBERSORT was used to assess the infiltration degree of immune cells. A total of 22 types of immune cells were evaluated: naïve/memory B cells, plasma cells, CD8+ T cells, naïve CD4+ T cells, resting/activated memory CD4+ T cells, follicular helper T cells, regulatory T cells (Treg cells), gamma delta T cells, resting/activated NK cells, monocytes, M0/M1/M2 macrophages, resting/activated dendritic cells, resting/activated mast cells, eosinophils, and neutrophils.

### Quantitative real-time polymerase chain reaction assay

Total RNA was extracted using TRIzol (Lot A2A0209, Accurate Biotechnology, Hunan Province, China) reagent and assessed using a Nanodrop 2000 system (Thermo Fisher Scientific, Waltham, MA, USA). A reverse transcription kit (A2A1386) was obtained from Accurate Biotechnology (Human) Co., China. The sequence of 18S, TBX5, and TBX5-AS1 primers is listed in Table S2. The real-time polymerase chain reaction (PCR) was performed on a LightCycler 480 II (Roche, Basel, Swiss), using the SYBR Green system (Lot A2A1436, Accurate Biotechnology, Hunan Province, China).

### Prediction of response to ICI treatment

Immunophenoscore (IPS) was applied to The Cancer Immunome Atlas (TCIA, https://tcia.at/) to quantify tumor immunogenicity and predict patients’ response to ICI treatment (Charoentong et al., 2017). The T-cell immune dysfunction and exclusion (TIDE) was used to predict whether patients could benefit from ICIs based on the cytotoxic T lymphocyte (CTL) status (Fu et al., 2020b). The preparation files were obtained from UCSC and normalized.

### The construction of competing endogenous RNA (ceRNA) network and prediction of combination region of TBX5-AS1 to AR

We used Starbase (https://starbase.sysu.edu.cn/) to predict potential target miRNA of TBX5-AS1 and miRDB (http://mirdb.org/) for AR. Besides, the catRAPID (http://service.tartaglialab.com/page/catrapid_group) was performed to explore the possibility of TBX5-AS1 combining AR.

### Statistical analysis

All R packages were run under the environment of R 3.6.1. GraphPad Prism (version 8.0) and SPSS 24.0 (IL, USA) were used to perform statistical analyses. A *p* value < 0.05 indicated a statistically significant difference.

## Result

### Contradictory effects of TBX5-AS1 in pan-cancer survival analyses

**Figure 1:**
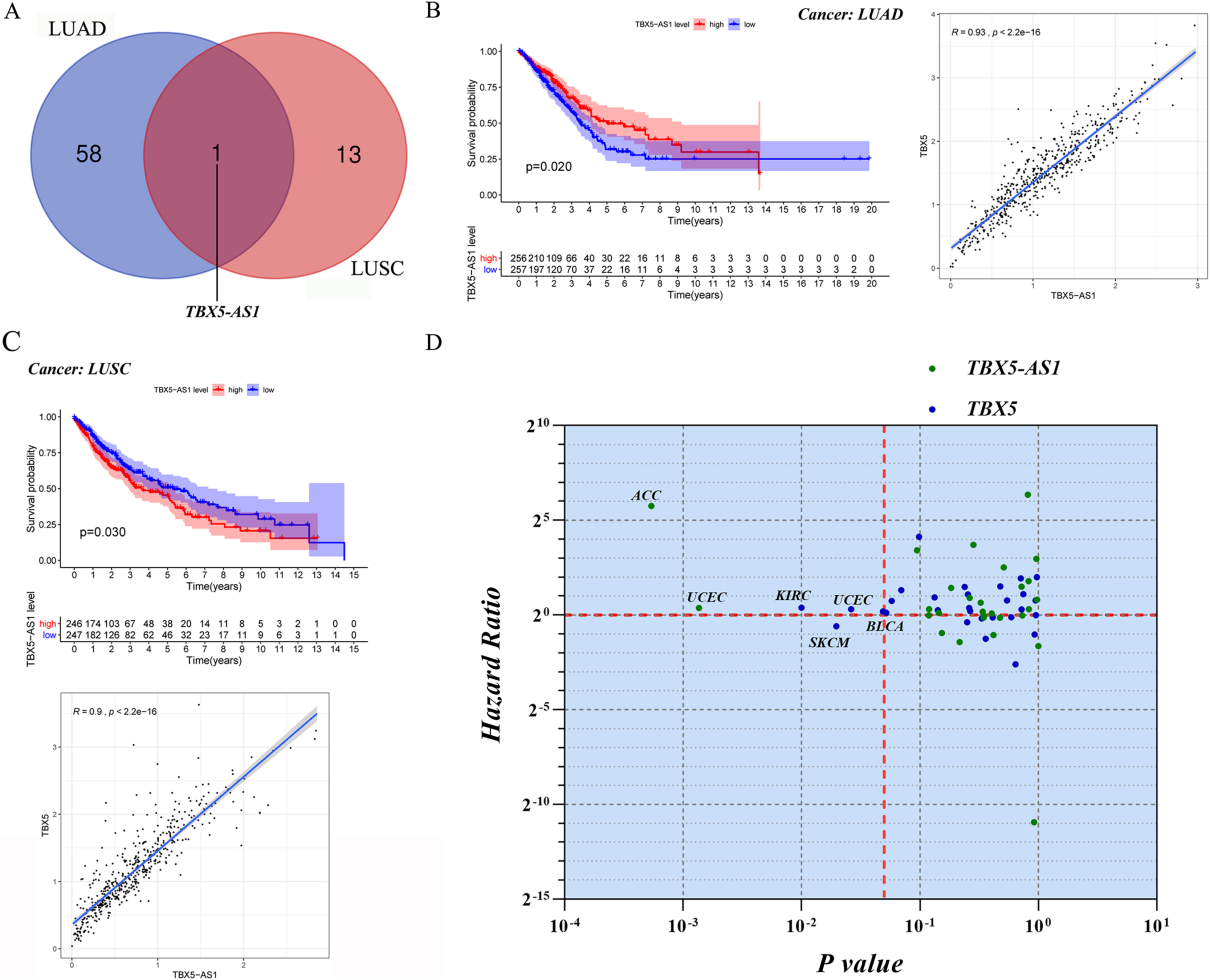
The screening process and survival prediction of TBX5-AS1. (A) Venn diagram showed that TBX5-AS1 was the only eRNA with prognostic significance for lung adenocarcinoma (LUAD) and lung squamous cell carcinoma (LUSC). (B) The protect role of TBX5-AS1 for LUAD (*n* = 513) and the strong correlation to homological target TBX5. (C) The risk role of TBX5-AS1 for LUSC (*n* = 493) and the strong correlation to TBX5. (D) Pan-cancer analysis based on TBX5-AS1 and TBX5 expression level screened the significant cancer types: adrenocortical carcinoma (ACC) and uterine *corpus* endometrial carcinoma (UCEC) for TBX5-AS1; kidney renal clear cell carcinoma (KIRC), bladder urothelial carcinoma (BLCA), skin cutaneous melanoma (SKCM) and UCEC for TBX5. The horizontal red line signs Hazard ratio is equal to one and the vertical red line marks *p* value equal to 0.05.

Screening of eRNAs with prognostic significance was initially performed in an NSCLC cohort (Tables 1 and 2). The Venn diagram showed that TBX5-AS1 might play a key role in the prognosis of patients with ADC and SCC (Fig. 1A), despite its equally strong correlation to target TBX5 in both NSCLC subtypes (*R* > 0.9 and *p* < 0.0001). Interestingly, TBX5-AS1 had opposite effects on the prognosis (Figs. 1B and 1C). TBX5-AS1 seemed to act as a risk factor for patients with SCC and became a protective factor in patients with ADC.

**Table 1:** The significant eRNAs in LUAD patients.

eRNA	KM	Target	cor	corPval
A2MP1	0.0016518	A2M	0.4569769	1.68E−28
		PZP	0.5409163	2.63E−41
AC007255.1	0.0032732	PRR15	0.8808246	2.74E−172
AC008957.1	0.0139699	SLC1A3	0.5384108	7.19E−41
AC012618.3	0.0193707	ZNF563	0.4621955	3.38E−29
AC025871.2	0.0053137	FBXO16	0.4260372	1.31E−24
AC027117.1	0.0166444	MTUS1	0.6525713	3.90E−65
AC090023.2	0.0141453	HMGA2	0.486134	1.49E−32
	0.0141453	RPSAP52	0.548231	1.34E−42
AC090559.1	0.000513	SPI1	0.7825607	0
AC091849.2	0.0054	LPCAT1	0.7101183	6.99E−82
		SDHAP3	0.5398715	4.01E−41
AC105942.1	0.0397474	CNN3	0.6328898	0
AC124242.1	0.00519	ASAH1	0.6347887	0
AL031846.1	0.0343209	APOBEC3C	0.4434384	9.52E−27
		APOBEC3D	0.6041216	1.24E−53
		APOBEC3F	0.4365803	6.87E−26
		APOBEC3G	0.6952433	3.58E−77
		APOBEC3H	0.6359948	5.73E−61
AL035670.1	0.0035174	RCAN2	0.5002838	1.16E−34
AL035701.1	0.0453327	ENPP5	0.5200788	8.69E−38
		ENPP4	0.6075	2.26E−54
AL136369.2	0.0348284	CELF2	0.4720267	1.53E−30
		SFTA1P	0.5205479	7.29E−38
AL138767.3	0.0416409	PAPSS2	0.5549796	8.00E−44
AL390778.2	0.0425501	OLFM1	0.4155737	2.22E−23
AP000424.1	0.0468313	RNF19A	0.4953191	6.54E−34
AP001347.1	0.0222727	RBM11	0.4192669	8.29E−24
AP001972.3	0.0099719	SLCO2B1	0.457574	1.40E−28
AP002992.1	0.0362573	CHKA	0.5279883	4.29E−39
AP003472.1	0.041767	RNF19A	0.4265516	1.14E−24
AP004608.1	0.0335144	B3GAT1	0.4621646	3.42E−29
AP5B1	0.0342212	SART1	0.4786506	0
BAALC-AS1	0.0446312	FZD6	0.6930719	0
C5orf66	0.0283318	PITX1	0.4521315	7.28E−28
CDK6-AS1	0.0406382	CDK6	0.4979558	2.62E−34
CHRNA1	0.0287456	CHRNA1	1	0
CRNDE	0.0008444	IRX5	0.8009783	0
CROCCP2	0.0237995	NBPF1	0.50358	0
GAS1RR	0.00058	GAS1	0.6871142	1.02E−74
HAGLR	0.0307416	HOXD1	0.8936992	1.58E−184
		HOXD3	0.4703208	2.63E−30
IFNG-AS1	0.0360017	IFNG	0.4374696	5.33E−26
JPX	0.0214615	XIST	0.5316121	1.05E−39
LINC00261	0.0468338	FOXA2	0.9018119	4.45E−193
LINC00460	0.0386313	EFNB2	0.5160698	3.88E−37
LINC00987	0.0060574	A2M	0.6573031	0
		PZP	0.5082778	6.70E−36
LINC00996	0.0045767	GIMAP4	0.7074833	0
		GIMAP6	0.6650255	0
		GIMAP7	0.6915918	0
		GIMAP8	0.6673479	0
LINC01031	0.0002358	B3GALT2	0.4343513	1.29E−25
LINC01088	0.0468212	NAA11	0.4405871	2.18E−26
LINC01615	0.0397124	THBS2	0.736348	5.88E−91
LINC01798	0.0158747	MEIS1	0.6734206	9.17E−71
LINC01833	0.0002146	SIX3	0.6407066	3.98E−62
LINC02390	0.0028058	CD69	0.4025137	6.62E−22
		CLECL1	0.4580082	1.23E−28
LINC02611	0.0042115	MGAT4A	0.4699959	0
LINC02705	0.0359998	MS4A6A	0.4579996	1.23E−28
LNCAROD	0.031311	DKK1	0.4193355	8.14E−24
LRRC8C-DT	0.020728	LRRC8C	0.7191624	0
MIR646HG	0.0280195	C20orf197	0.6830398	1.61E−73
		CDH26	0.5002143	1.19E−34
NBPF1	0.0098521	CROCCP2	0.50358	0
OGFRP1	4.36E-06	TCF20	0.4622622	0
PCBP1-AS1	0.0321381	ASPRV1	0.5221949	0
		TIA1	0.65801	0
PRDM16-DT	0.0002991	PRDM16	0.9021302	1.98E−193
PRKG1-AS1	3.56E-05	DKK1	0.7241655	1.30E−86
SKINT1L	0.0445155	SLC5A9	0.4993538	1.60E−34
SLC2A1-AS1	0.0107498	SLC2A1	0.5761279	0
SOX2-OT	0.0167841	SOX2	0.7545271	0
TBX5-AS1	0.0198774	TBX5	0.9252466	0
WT1-AS	0.0464188	WT1	0.8740294	2.18E−166

**Table 2:** The significant eRNA in LUSC patients.

eRNA	KM	Target	cor	corPval
LINC01714	0.0016131	ERRFI1	0.4164722	1.94E−22
LINC01121	0.02078	SIX2	0.5492531	7.99E−41
AC005042.2	0.0385368	DAPL1	0.508111	3.05E−34
LINC01280	0.0212273	IGFBP2	0.6013095	1.40E−50
AC092919.2	0.035896	TNIK	0.6274091	3.50E−56
LINC02068	0.0118051	TNFSF10	0.4752438	1.36E−29
LINC02043	0.0495869	AHSG	0.4624998	6.39E−28
		FETUB	0.4697411	7.31E−29
CASC11	0.0387377	PVT1	0.4684339	1.09E−28
AC079209.1	0.0479611	PAG1	0.4855658	5.34E−31
CYP4F26P	0.0227265	SUGT1P1	0.4247519	2.30E−23
MIR100HG	0.0327234	MIR100HG	1	0
TBX5-AS1	0.0299848	TBX5	0.8997356	0
OTX2-AS1	0.0095065	OTX2	0.593494	5.32E−49
APCDD1L-DT	0.005061	APCDD1L	0.7885642	1.94E−107

For further pan-cancer analysis, the prognostic values of TBX5 and TBX5-AS1 were analyzed in 31 other solid cancers from the TCGA database *via* hazard ratio (HR) calculation and KM *p* value. The data were scattered in four quadrants, split by *p* = 0.05 and HR = 1. In ACC and uterine *corpus* endometrial carcinoma (UCEC) cohorts, the expression level of TBX5-AS1 was associated with worse outcomes. The same was true for TBX5 in kidney renal clear cell carcinoma, UCEC, and bladder urothelial carcinoma (Fig. 1D). On the contrary, patients with skin cutaneous melanoma having high expression of TBX5 might possess a better prognosis.

### Specific connection between TBX5/TBX5-AS1 and gender

**Figure 2:**
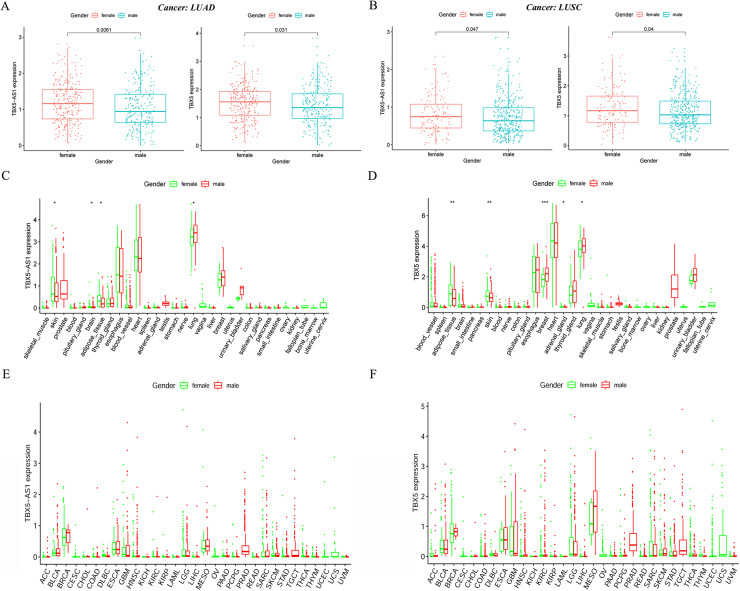
The expression difference of TBX5 and TBX5-AS1 between male and female. The expression level of TBX5-AS1 and TBX5 of female patients was higher than male in LUD (A) and LUSC (B). The distribution difference of TBX5-AS1 (C) and TBX5 (D) expression in normal human organs between male and female. Meanwhile, the distribution difference of TBX5-AS1 (E) and TBX5 (F) expression in multiple cancer cohorts based on gender group. “*” means *p* < 0.05. “**” means *p* < 0.01. “***” means *p* < 0.001.

In the LUAD cohort, the expression levels of TBX5 and TBX5-AS1 were influenced by gender (Fig. 2A). Similarly, the expression levels of TBX5 and TBX5-AS1 in male patients with SCC were significantly lower than those in female patients (Fig. 2B). However, there was no difference of TBX5-AS1 and TBX5 expression based on age grouping (Fig. S1). The analysis of organ gene expression based on the GTEx database showed that TBX5 and its eRNA were mainly enriched in the heart and lungs of the normal population (Fig. S2). After distinguishing gender as a variable, TBX5-AS1 and TBX5 were expressed differently in the lung of male and female patients (Figs. 2C & 2D). Different distributions of TBX5-AS1 between male and female patients occurred in the skin, brain, and adipose tissue. A similar distribution of TBX5 also existed in the skin, adipose tissue, breast, and adrenal gland. That indicated eRNAs with targets might have tissue and gender specificity (Fig. S2). eRNAs might played different roles in male and female. Besides, the pan-cancer screen predicted that this expression difference between female and male did not appear in other cancer cohorts of TCGA database (Figs. 2E & 2F).

### Survival analysis of male and female patients in NSCLC and cohort validation

**Figure 3:**
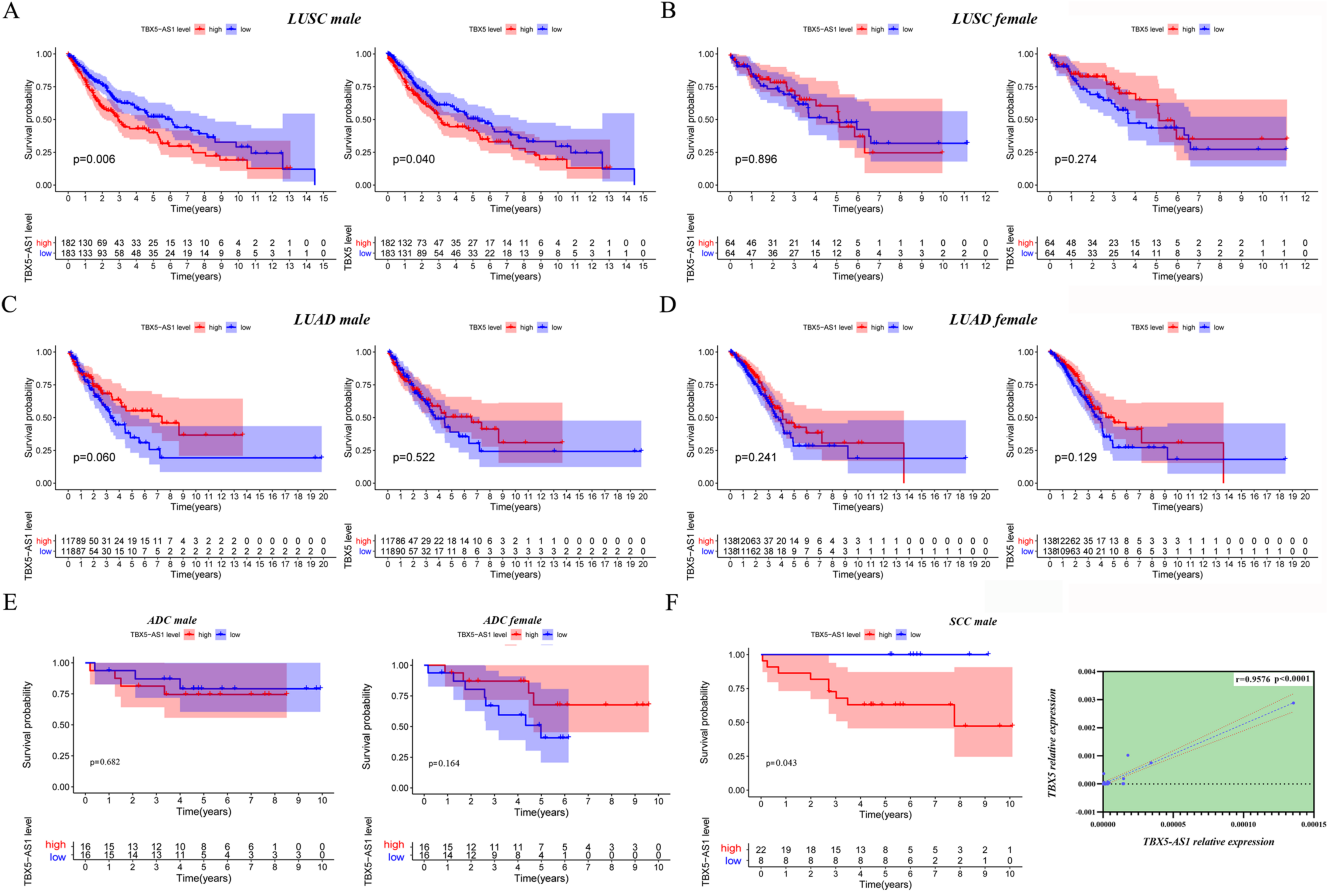
The survival analysis based on TBX5 and TBX5-AS1 expression after gender grouping. The KM plot for TBX5 and TBX5-AS1 expression in male LUSC patients (*n* = 365, A), female LUSC patients (*n* = 128, B), male LUAD patients (*n* = 237, C) and female LUAD patients (*n* = 276, D) from TCGA cohorts. (E) The KM plot for validation cohort including male patients with ADC (locating at left) and female patients with ADC (locating at right) from Shandong Province Hospital based on TBX5-AS1 expression. (F) The KM plot for validation cohort including male patients (*n* = 30) with SCC and the correlation between RNA expression level of TBX5 and TBX5-AS1 in validation cohort from Shandong Province Hospital.

Given that the gender could cause TBX5 and TBX5-AS1 lung research bias, the KM curves were plotted in male and female patients separately. Interestingly, the significant results of TBX5 and TBX5-AS1 converged at the male LUSC cohort (*p* = 0.006 for TBX5-AS1 and 0.040 for TBX5; Fig. 3A). The prognosis of the rest of the groups, female LUSC, male LUAD, and female LUAD, were not significantly associated with TBX5 or TBX5-AS1 (Figs. 3B–3D).

Moreover, 32 female patients with ADC, 32 male patients with ADC, and 30 male patients with SCC were included to form a validation cohort. The survival analysis proved no significant difference between low- and high-expression groups of TBX5-AS1 in the female and male ADC cohorts (Fig. 3E). The prognosis was better in the low-expression group of TBX5-AS1 compared with the high-expression group in male patients with SCC, and the quantitative reverse transcriptase (qRT)-PCR verified that a strong correlation existed between eRNA and its homologous target once again (*r* = 0.9576 and *p* < 0.0001; Fig. 3F).

**Figure 4:**
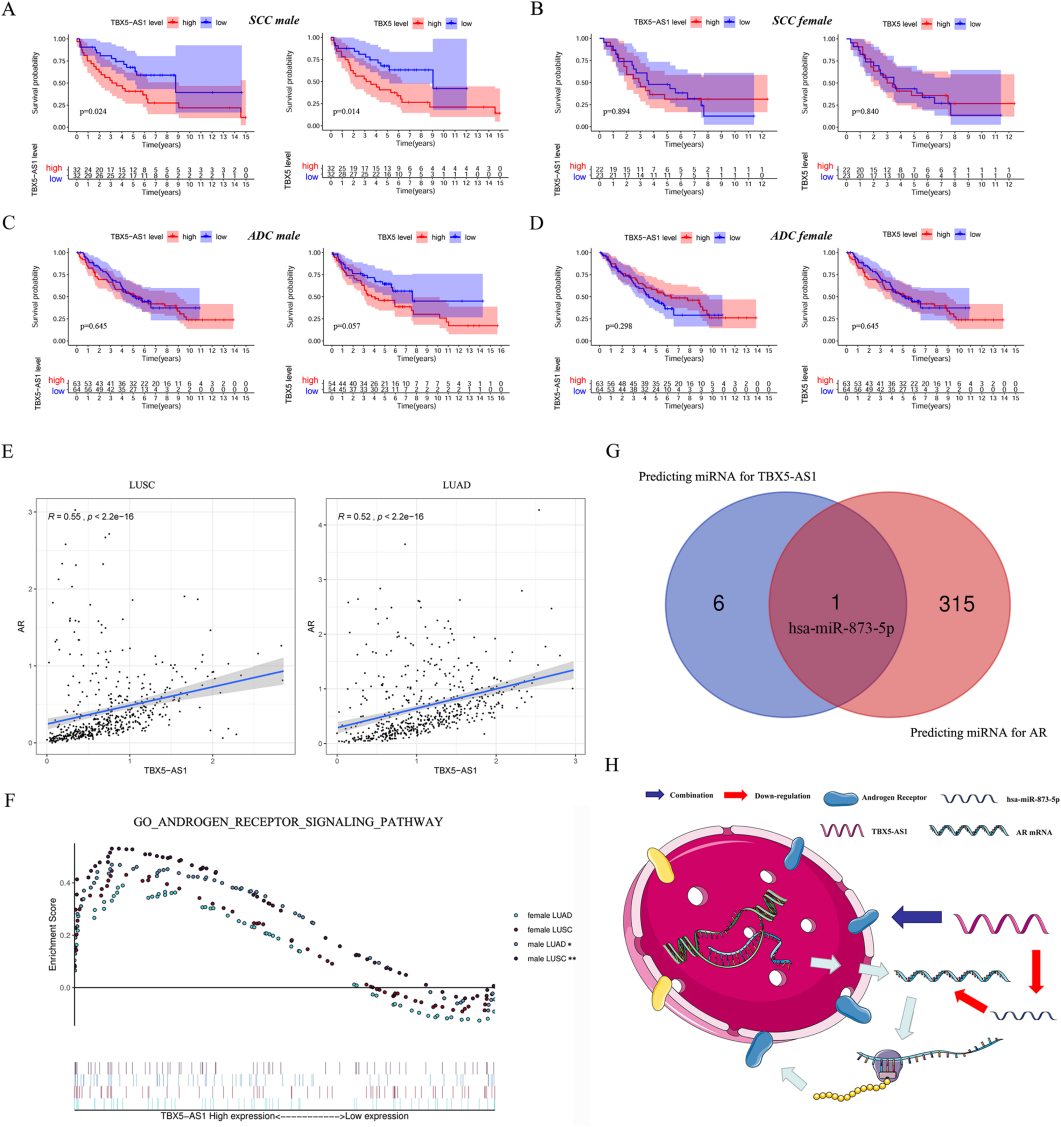
The survival analysis based on TBX5 and TBX5-AS1 expression in GEO cohorts and the correlation between androgen signal pathway and TBX5-AS1. The KM plot for TBX5 and TBX5-AS1 expression in male SCC patients (A), female SCC patients (B), male ADC patients (C) and female ADC patients (D) from GSE37745 and GSE50881. (E) The expression of TBX5-AS1 and AR at RNA level was positive related in LUSC and LUAD. (F) Verification of the relationship between TBX5-AS1 expression and activation level of androgen signal pathway *via* GSEA for LUAD and LUSC patients. According to sequence of female LUAD, female LUSC, male LUAD and male LUSC, the FDRs were as follows: 0.079, 0.088, 0.021, 0.000; and the *p* values were as follows: 0.079, 0.088, 0.021, 0.000; the NESs were as follows: 1.54, 1.58, 1.73, 2.02. (G) The Venn diagram based on Starbase and miRDB database predicted hsa-miR-873-5p was the potential candidate to build ceRNA network. (H) The potential mechanism of TBX5-AS1 regulating AR expression *via* ceRNA network and combination between TBX5-AS1 and AR. “*” means *p* < 0.05. “**” means *p* < 0.01.

Furthermore, the gender specificity of TBX5 and TBX5-AS1 in patients with NSCLC was validated by the combined cohorts based on GSE37745 and GSE50881. The TBX5-AS1 (*p* = 0.024) and TBX5 (*p* = 0.014) exerted a prognostic biomarker effect only in male patients with SCC (Fig. 4A). For female patients with NSCLC and those with SCC, the result was not statistically significant (Figs. 4B–4D).

### Correlation between TBX5-AS1 and AR signaling pathway

Considering the vast impact of gender differences on the role of TBX5-AS1, a correlation analysis between the expression levels of TBX5-AS1 eRNA and AR mRNA was performed in LUSC and LUAD samples. For male patients, the correlation was evident: *R* = 0.55 in LUSC and 0.52 in LUAD (both *p* < 0.0001; Fig. 4E). Furthermore, the GSEA results indicated that the androgen-mediated pathway (GO_ANDROGEN_RECEPTOR_SIGNALING_PATHWAY) was enriched in the male LUSC (*p* = 0.002) and male LUAD (*p* = 0.021; Fig. 4F) groups. For female patients, the outcomes were not significant (*p* = 0.079 for LUAD and *p* = 0.088 for LUSC). Based on miRDB and Starbase databases, we performed Venn diagram and found hsa-miR-873-5p might become the important bridge to connect ceRNA network of TBX5-AS1 and AR (Fig. 4G). Meanwhile catRAPID was used to predict the combination region between TBX5-AS1 and AR, under the consideration of classic interaction of eRNAs and hormone receptors (Table S3). Thus, the schematic diagram was showed in Fig. 4H to exhibited that TBX5-AS1 could stabilized the expression of AR *via* ceRNA network and interact with AR to influence downstream pathways.

### Predicting prognosis in male and female pan-cancer cohorts

**Figure 5:**
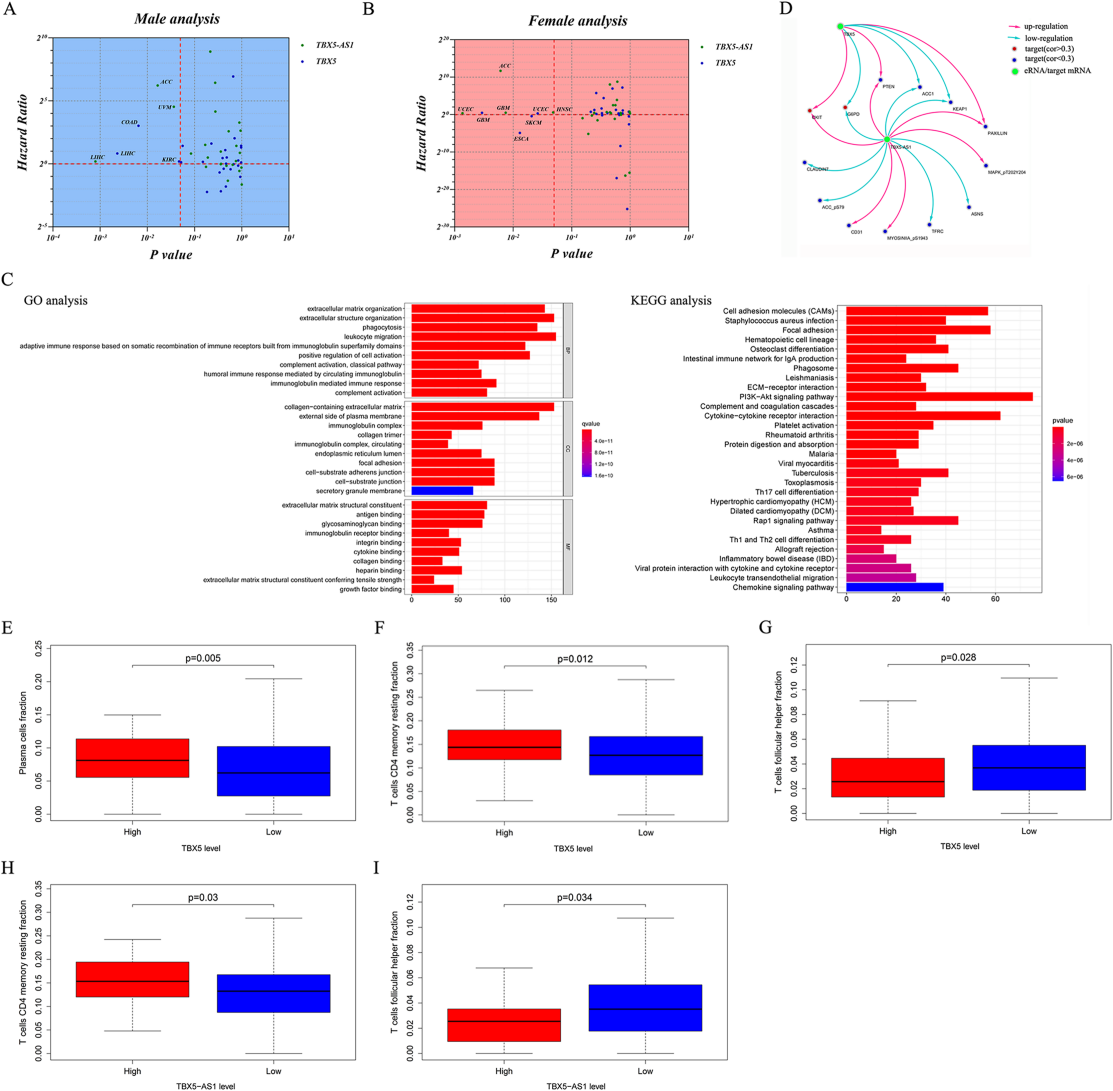
Pan-caner analysis, functional analyses and evaluation of immune microenvironment. Pan-cancer prognostic analysis for 31 types solid cancers based TBX5 and TBX5-AS1 in male (A) and female (B) patients. The horizontal red line signs Hazard ratio is equal to 1 and the vertical red line marks *p* value equal to 0.05. (C) The enrichment of GO term and KEGG pathway based on genes correlated to TBX5-AS1. (D) The prediction of potential protein targets to TBX5-AS1 and TBX5. The infiltrated fraction of plasma (E) and T cell CD4 memory resting (F) significantly increased in the high expression group of TBX5, while T cell follicular cell (G) fraction decreased. The same situation of T cell follicular cell (H) and T cell CD4 memory resting (I) occurred in the high expression group of TBX5-AS1.

Figure 1D shows the prognostic significance of TBX5-AS1 in ACC and UCEC cohorts, and the consistency in predicting prognosis between TBX5-AS1 and TBX5 existed only in female UCEC samples. Thus, given the connection between TBX5-AS1 and gender, the pan-cancer survival analysis was performed after gender grouping. In various cancers, the prognostic significance of TBX5-AS1 and its homologous target became more consistent, which were the risk factors for liver hepatocellular carcinoma (LIHC) in male patients and glioblastoma multiforme (GBM) and UCEC in female patients (Figs. 5A and 5B). Additionally, a significant prediction was explored for both TBX5-AS1 and TBX5.

### Functional analyses of TBX5-AS1 in male LUSC samples

The KEGG and GO analyses were performed based on the correlated genes of TBX5-AS1 (*R* > 0.4). The immune-related terms, “leukocyte migration,” “adaptive immune response based on somatic recombination,” and “humoral immune response mediated by circulating immunoglobin,” were significant in GO analyses (Fig. 5C). Similarly, the result of KEGG analysis also indicated that the impact of TBX5-AS1 on cell biology might focus on immunological pathways, for example, “Th17 cell differentiation,” “leukocyte transendothelial migration,” and “Th1 and Th2 cell differentiation” (Fig. 5C).

Target prediction at the protein level based on TCPA databases indicated that CKIT, PTEN, and PAXILLIN were positively related to TBX5-AS1 and TBX5, and the correlated relationship to the targets ACC1, G6PD, and KEAP1 was negative (Fig. 5D). Besides, the potential targets individually to eRNA were listed as follows and (−)/(+) meant negative/positive correlation: CLAUDIN7 (−), ACC_pS79 (−), CD31 (+), MYOSINIA_pS1943 (+), TFRC (−), ASNS (−), and MAPK_pT202Y204 (+).

### Evaluation of infiltrated immune cells in male patients with LUSC

The infiltrated fraction of plasma cell and resting memory CD4 T cells increased in the high-expression group of TBX5 (Figs. 5E and 5G). Meanwhile, the situation in follicular helper T cells was the opposite (Fig. 5F). For TBX5-AS1, the connection to resting memory CD4 T cells and follicular helper T cells was similar to that for TBX5 (Figs. 5H and 5I).

The correlated heatmap exhibited that the infiltrated fraction of resting memory CD4 T cells was connected with the immunological infiltration of CD8 T cells (−0.56), activated CD4 T cells (−0.37), and follicular helper T cells (−0.36; Fig. S3), which supported the aforementioned results.

### Prediction of response to ICI treatment according to the expression of TBX5-AS1

**Figure 6:**
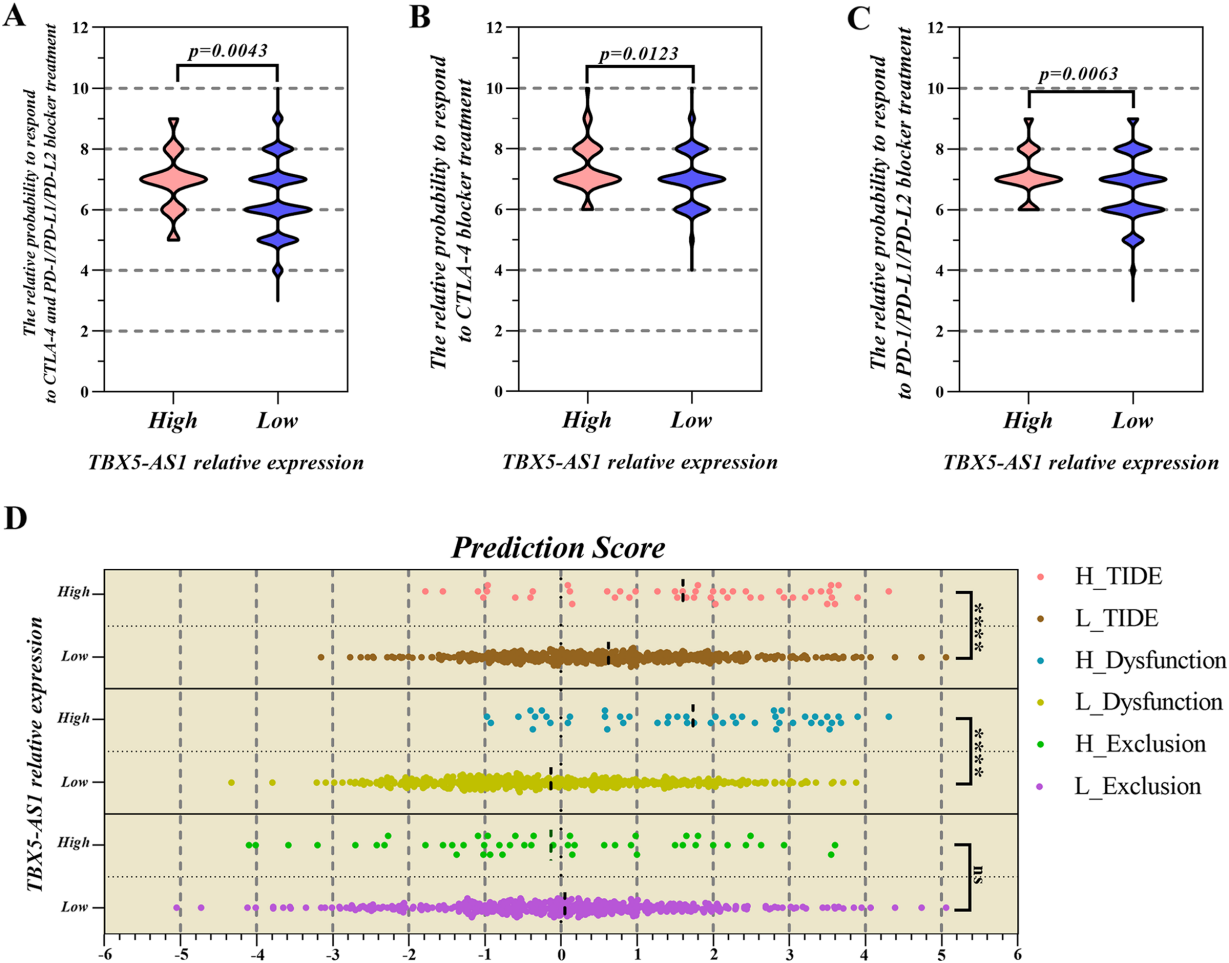
The prediction of response to immune checkpoint inhibitors (ICIs) treatment. The PSI value of PD1 inhibitors (A), CTLA-4 inhibitors (B) and combination therapy (C) was significantly higher in high expression group of TBX5-AS1 than low expression group in LUSC patients. (D) The microenvironment evaluation based on TIDE indicated that the TIDE score and T cell dysfunction score was higher in high expression level group of TBX5-AS1 than low expression group. “****” means *p* < 0.0001.

Based on the consideration that TBX5-AS1 influenced the T lymphocyte function, its role in response to lung squamous cancer immunotherapy was further explored (Waldman, Fritz & Lenardo, 2020). The IPS files of SCC patients were downloaded from the TCIA database, and the high value suggested that patients were sensitive to immunotherapy. The IPS of programmed cell death protein 1 (PD1) inhibitors was higher in the high-expression group of TBX5-AS1 (mean = 7.146) than in the low-expression group (mean = 6.704; Fig. 6A). The IPS of the Cytotoxic Lymphocyte Antigen 4 (CTLA-4) blocker was higher in the high-expression group of TBX5-AS1 (mean = 7.354) than in the low-expression group (mean = 7.022; Fig. 6B). As for the combination therapy of CTLA-4 blockers and PD1 inhibitors, the IPS was higher in the high-expression group of TBX5-AS1 (mean = 6.917) than in the low-expression group (mean = 6.404; Fig. 6C). All results were statistically significant.

The TIDE algorithm focused on the malignant degree of the tumor microenvironment (TME) (Fu et al., 2020b). When infiltrated CTLs were rare, the TIDE score was equal to the T-cell exclusion score and also to T-cell dysfunction in the high-CTL situation. Figure 6D shows that patients with high expression of TBX5-AS1 might possess worse TME, which reflected in the T-cell dysfunction (mean of high expression = 1.735 and mean of low expression = −0.331; *p* < 0.0001).

Because TIDE algorithm was developed for lung cancer and melanoma, we only used IPS to predict the immunotherapy responses in male patients with LIHC and female patients with GBM and UCEC. In addition to meaningless in GBM cohort, the outcome in UCEC and LIHC cohorts was significant but opponent to result in SCC patients (Table S4). In this situation, patients with low TBX5-AS1 expression might are sensitive to immunotherapy including PD1 inhibitors, CTLA-4 blockers and combination therapy of both them.

## Discussion

The emerging of eRNAs is the landmark for research of transcriptional regulation, and numerous oncological studies have been published (Gu et al., 2019; Mao et al., 2019; Zhang et al., 2020; Pan et al., 2021; Wang et al., 2021). Zhang found that eRNAs were correlated with 80.8% of 229 cancer signaling genes (cell cycle, Hippo, Notch, PI3K, Nrf2, RAS, p53, etc) (Zhang et al., 2019). Seldom eRNAs are recognized as targets for diagnosis and treatment and put into clinical practice until now. For example, SCRIBe, the eRNA associated with oncogene SCRIB, was found to be differentially expressed among lung adenocarcinoma patients according to smoking history (Zhang et al., 2019). However, although nascent RNA sequencing has removed shackles in the technology for exploring eRNAs, detailed research is not fully developed (Kristjansdottir et al., 2020; Goodall & Wickramasinghe, 2021). The key role of eRNAs in estrogen and androgen signaling pathways suggested that gender differences, which significantly influenced the prognosis or immune system in multiple types of carcinomas, might be one of the underlying mechanisms (Hah et al., 2013; Hsieh et al., 2014; Radkiewicz et al., 2017; Takahashi & Iwasaki, 2021). Besides, we especially agree with Wilson, M. A. and Buetow, K. H. that gender should be treated as a biological variable in all researches, but unfortunately, most studies involving “cancer” and “TCGA” did not include gender (Wilson & Buetow, 2020). The findings of the present study revealed that the expression level of TBX5-AS1 eRNA in various organs was related to gender in normal people and patients with carcinoma. Furthermore, this characteristic was the important cause of bias in survival analysis. Therefore, this study focused on the key role of TBX5-AS1 in gender differences, prognosis, and immunology.

It must be admitted that the appearance of eRNA TBX5-AS1 was dramatic. eRNAs with prognostic significance were screened in ADC and SCC cohorts. The Venn diagram showed that the intersection of the two groups contained only TBX5-AS1, but its prognostic efficiency was the opposite for ADC and SCC (Figs. 1B and 1C). First, the discovery of a critical molecule that revealed the difference between subtypes of NSCLC was convincing. However, the significant difference in the expression level of TBX5-AS1 between male and female patients indicated that the previous conclusion might be biased (Figs. 2A and 2B). Furthermore, this gender difference existed in even normal organs, such as the skin, brain, adipose tissue, lung, and urinary bladder. The extensiveness undoubtedly indicated that gender should be regarded as a biological variable to limit, which was a prerequisite for exploring TBX5-AS1 function.

The eRNA TBX5-AS1 and its homologous target TBX5 were risk factors for male patients with SCC, and the synergistic effect was absent in other groups: female patients with SCC and male/female patients with ADC. Besides, the pan-cancer analysis also added positive results after gender grouping. For male patients, the expression levels of TBX5 and TBX5-AS1 influenced the prognosis of LIHC. For female patients, UCEC and GBM cohorts seemed to suffer from the increased expression levels of TBX5-AS1 and its homological target. The findings of this study reminded us of the indispensability of gender differences in the TCGA analysis.

“TBX5-AS1” was searched through PubMed, and eight records related to NSCLC (divided into two groups, ADC and SCC), neuroblastoma, glioblastoma, Tetralogy of Fallot, and bat wing development, were obtained (Eckalbar et al., 2016; Qiao, Li & Li, 2018; Xiong et al., 2019; Ma et al., 2020; Niu et al., 2020; Qu, Jiang & Li, 2020; Shih et al., 2020; Ye et al., 2020). TBX5-AS1 was recognized as a risk factor in GBM from the Chinese Glioma Genome Atlas and TCGA databases, which was in line with the result of prognostic analysis in female patients with GBM (Fig. 5D) (Niu et al., 2020). For nonsmoking female patients with ADC and lung cancer, TBX5-AS1 exerted a protective role in prognosis prediction (Qiao, Li & Li, 2018; Shih et al., 2020). Conversely, Zhang W and his team found that the high-expression level of TBX5-AS1 was associated with an unfavorable prognosis (Xiong et al., 2019). This contradictory phenomenon was the same as the initial result.

In a functional analysis for male patients with SCC, the RNA expression level of both TBX5-AS1 and TBX5 had a negative correlation with the KEAP1 protein abundance. As the classic anti-oncogene, KEAP1 could inhibit tumor occurrence and metastasis *via* interacting with oncogene NRF2 (Hamada et al., 2021). Meanwhile, KEAP1 mutation was a therapeutic target for Telaglenastat, the new targeted drug for NSCLC, which was expected to bring new hope to more than 20% of patients with NSCLC (Riess et al., 2021). Moreover, the potential protein targets of TBX5-AS1 were far more than those of TBX5, indicating that the downstream effects of TBX5-AS1 were not limited to its homological mRNA. For example, the protein abundance of phosphate MAPK (T202/Y204) increased following the expression of TBX5-AS1, which induced the activation of the ERK pathway and mesenchymal transition (Lai & Pelech, 2016; Nahomi, Pantcheva & Nagaraj, 2016). In addition to the common protein target with TBX5, such as CKIT, PTEN, PAXILLIN, ACC1, G6PD, and KEAP1, the potential downstream of TBX5-AS1 also included CLAUDIN7 (−), ACC_pS79 (−), CD31 (+), MYOSINIA_pS1943 (+), TFRC (−), ASNS (−), and MAPK_pT202Y204 (+). The huge and comprehensive regulatory network is a powerful capital for TBX5-AS1 to become a novel therapeutic target.

Given the considerable difference in the immune status between female and male patients, immunotherapy strategies varied for both sexes (Wang, Cowley & Liu, 2019). As a gender-specific eRNA, TBX5-AS1 might exert a substantial effect on the immune microenvironment. The KEGG and GO analyses pointed out the relationship between TBX5-AS1 and immunology. The evaluation of infiltrated immune cells indicated that the infiltrated fraction of T-cell memory resting rose up in the high-expression group of TBX5-AS1. On the contrary, the number of recruited follicular T cells dropped. The correlation heatmap indicated that the resting memory T cells negatively influenced CD8 T cells (correlation value = −0.56), being one of the crucial factors for benign immune microenvironment construction, and profoundly affected the therapeutic effect of ICI treatment (Joyce & Fearon, 2015; Jiang et al., 2018).

Multiple ICI treatments are inevitably accompanied by specific immune-related adverse events, including colitis, pneumonitis, and interstitial lung disease. Therefore, choosing appropriate immune inhibitors for patients is crucial (Chen et al., 2021). The high expression of TBX5-AS1 represented an unfavorable ending for male patients with SCC. The assessment of tumor microenvironment demonstrated that T-cell dysfunction was exacerbated in the increased expression group based on TIDE. Fortunately, IPS prediction indicated that ICI treatment response, including PD1 inhibitors, CTLA-4 blockers, and combination therapy, was more favorable in the high-expression group of TBX5-AS1. The TIDE and IPS results suggested that TBX5-AS1 could provide a reference for immunotherapy clinically. However, for male patients with LIHC and female patients with UCEC, low expression level of TBX5-AS1 mean sensitivity to immunotherapy, which indicated that TBX5-AS1 might play variety roles in multiple cancers.

The GSEA-based functional enrichment analysis proved that the expression level of TBX5-AS1 was influenced by the activation of the androgen signaling pathway in male patients. Given the classic regulation mode in published studies, the expression level of TXB5-AS1 was likely to increase at the transcription level after the androgen signaling pathway and play a key role in regulating downstream molecules to influence multiple biological behaviors as immune response. Existing literatures show that eRNAs always combined AR or ER to exert effects (Hah et al., 2013; Hsieh et al., 2014), hence we used catRAPID and found TBX5-AS1 possessed multiple regions to contact with AR (Table S3). Based on the established ceRNA network, we proposed hypothesis that TBX5-AS1 might combine AR to influence downstream pathway and obstruct miRNA hsa-miR-873-5p to upregulate AR expression. The traits of TBX5-AS1 integrated gender, hormones, and the immune system to form a complete logical axis, which laid the foundation for the present study.

Additionally, the importance of gender differences in oncology and bioinformatics needs to be further addressed. Inevitably, this study had some limitations. For example, basic experiments for biological behavior influenced by TBX5-AS1 were lacking. Besides, the downstream mechanism of TBX5-AS1 to regulate the progression of prognosis-related cancers is still unclear. Moreover, the biological significance and clinical value of TBX5-AS1 needed further exploration.

## Conclusions

The gender-specific TBX5-AS1 eRNA predicted poor prognosis for male patients with lung SCC and provided references to clinical ICI treatment. The gender grouping helped screen potential therapeutic targets more accurately for pan-cancer analysis.

## Supplemental Information

10.7717/peerj.12536/supp-1 Supplemental Information 1 The list of solid cancer types in the article.

10.7717/peerj.12536/supp-2 Supplemental Information 2 The Primer sequence in research.

10.7717/peerj.12536/supp-3 Supplemental Information 3 The prediction of the combination region between TBX5-AS1 and AR.

10.7717/peerj.12536/supp-4 Supplemental Information 4 The prediction of response to immunotherapy between low and high expression group of TBX5-AS1 in LIHC, UCEC and GBM cohorts.

10.7717/peerj.12536/supp-5 Supplemental Information 5 The expression difference of TBX5 and TBX5-AS1 between young (<65 years) and old (≥65 years) group.

10.7717/peerj.12536/supp-6 Supplemental Information 6 In the normal human body, the expression level of TBX5-AS1 (A) and TBX5 (B) enriched in lung and heart.

10.7717/peerj.12536/supp-7 Supplemental Information 7 The correlation heatmap of infiltrated immune cells.

## Additional Information and Declarations

### Data Availability

The following information was supplied regarding data availability: The raw data is available in the Supplemental Files.
