# Supplementary material for: Gender specific eRNA TBX5-AS1 as the immunological biomarker for male patients with lung squamous cell carcinoma in pan-cancer screening
Source: PeerJ. 2021 Nov 25;9:e12536. doi: 10.7717/peerj.12536 (PMC8627656; doi:10.7717/peerj.12536)
Supplement: Supplemental Information 1 [file peerj-09-12536-s001.docx]

| Supplementary Table 1 The list of soild cancaer types involved in the article. | |
| --- | --- |
| Cancer Type | Abbreviation |
| Adrenocortical carcinoma | ACC |
| Bladder Urothelial Carcinoma | BLCA |
| Breast invasive carcinoma | BRCA |
| Cervical squamous cell carcinoma and endocervical adenocarcinoma | CESC |
| Cholangiocarcinoma | CHOL |
| Colon adenocarcinoma | COAD |
| Lymphoid Neoplasm Diffuse Large B-cell Lymphoma | DLBC |
| Esophageal carcinoma | ESCA |
| Glioblastoma multiforme | GBM |
| Head and Neck squamous cell carcinoma | HNSC |
| Kidney Chromophobe | KICH |
| Kidney renal clear cell carcinoma | KIRC |
| Kidney renal papillary cell carcinoma | KIRP |
| Acute Myeloid Leukemia | LAML |
| Brain Lower Grade Glioma | LGG |
| Liver hepatocellular carcinoma | LIHC |
| Mesothelioma | MESO |
| Ovarian serous cystadenocarcinoma | OV |
| Pancreatic adenocarcinoma | PAAD |
| Pheochromocytoma and Paraganglioma | PCPG |
| Prostate adenocarcinoma | PRAD |
| Rectum adenocarcinoma | READ |
| Sarcoma | SARC |
| Skin Cutaneous Melanoma | SKCM |
| Stomach adenocarcinoma | STAD |
| Testicular Germ Cell Tumors | TGCT |
| Thyroid carcinoma | THCA |
| Thymoma | THYM |
| Uterine Corpus Endometrial Carcinoma | UCEC |
| Uterine Carcinosarcoma | UCS |
| Uveal Melanoma | UVM |
| Lung adenocarcinoma | LUAD |
| Lung squamous cell carcinoma | LUSC |
