# Supplementary material for: Gender specific eRNA TBX5-AS1 as the immunological biomarker for male patients with lung squamous cell carcinoma in pan-cancer screening
Source: PeerJ. 2021 Nov 25;9:e12536. doi: 10.7717/peerj.12536 (PMC8627656; doi:10.7717/peerj.12536)
Supplement: Supplemental Information 2 [file peerj-09-12536-s002.docx]

| Supplementary Table 2 The Primer sequence in research | |
| --- | --- |
| Primer | Sequence (5'→3'） |
| TBX5-F | GTACCTGCCGACGATCACAG |
| TBX5-R | CACGATGTGTAATCTAGGCTGG |
| TBX5-AS1-F | GAGGAGGTGAAGGGAGGTAGGT |
| TBX5-AS1-R | AGGAGACTGGCTGGAGAGGAA |
| 18SrRNA-F | AAACGGCTACCACATCCAAG |
| 18SrRNA-R | CCTCCAATGGATCCTCGTTA |
