# Supplementary material for: Gender specific eRNA TBX5-AS1 as the immunological biomarker for male patients with lung squamous cell carcinoma in pan-cancer screening
Source: PeerJ. 2021 Nov 25;9:e12536. doi: 10.7717/peerj.12536 (PMC8627656; doi:10.7717/peerj.12536)
Supplement: Supplemental Information 3 [file peerj-09-12536-s003.docx]

| Supplement Table 3 The prediction of the combination region between TBX5-AS1 and AR. | | | | | |
| --- | --- | --- | --- | --- | --- |
| rnaFrag_start | rnaFrag_end | Interaction_Propensity | Z_score | | Ranking |
| 641 | 692 | 42.81 | | 0.52 | 0.411504 |
| 466 | 517 | 34.5 | | 0.3 | 0.402368 |
| 701 | 752 | 31.98 | | 0.23 | 0.399598 |
| 616 | 667 | 30.47 | | 0.19 | 0.397938 |
| 676 | 727 | 24.99 | | 0.05 | 0.391913 |
| 716 | 767 | 24.66 | | 0.04 | 0.39155 |
| 691 | 742 | 17.85 | | -0.14 | 0.384063 |
| 516 | 567 | 16.18 | | -0.19 | 0.382227 |
| 441 | 492 | 13.53 | | -0.26 | 0.379314 |
| 476 | 527 | 8.02 | | -0.4 | 0.373256 |
| 1 | 52 | 0.13 | | -0.61 | 0.364582 |
| 601 | 652 | -3.14 | | -0.7 | 0.360987 |
| 101 | 152 | -3.31 | | -0.7 | 0.3608 |
| 326 | 377 | -6.38 | | -0.78 | 0.357425 |
| 316 | 367 | -10.63 | | -0.89 | 0.352753 |
| 126 | 177 | -10.68 | | -0.9 | 0.352698 |
| 376 | 427 | -14.22 | | -0.99 | 0.348806 |
| 591 | 642 | -14.87 | | -1.01 | 0.348091 |
| 91 | 142 | -16.82 | | -1.06 | 0.345948 |
| 241 | 292 | -20.39 | | -1.15 | 0.342023 |
| 216 | 267 | -21.19 | | -1.17 | 0.341143 |
| 66 | 117 | -21.56 | | -1.18 | 0.340737 |
| 276 | 327 | -28.66 | | -1.37 | 0.332931 |
| 166 | 217 | -34.05 | | -1.51 | 0.327005 |
| 151 | 202 | -35.66 | | -1.55 | 0.325235 |
| 266 | 317 | -42.74 | | -1.74 | 0.317452 |
