# Supplementary material for: Gender specific eRNA TBX5-AS1 as the immunological biomarker for male patients with lung squamous cell carcinoma in pan-cancer screening
Source: PeerJ. 2021 Nov 25;9:e12536. doi: 10.7717/peerj.12536 (PMC8627656; doi:10.7717/peerj.12536)
Supplement: Supplemental Information 4 [file peerj-09-12536-s004.docx]

| Supplement Table 4 The prediction of response to immunotherapy between low and high expression group of TBX5-AS1 in LIHC, UCEC and GBM cohorts. | | | | |
| --- | --- | --- | --- | --- |
| Cancer Type | Immunotherapy | IPS values (Low expression) | IPS values (High expression) | P values |
| LIHC | PD1 inhibitors | 6.982 | 7.408 | 0.0146 |
|  | CTLA-4 blockers | 7.945 | 8.34 | 0.0089 |
|  | Combination theraoy | 6.618 | 6.984 | 0.0425 |
| UCEC | PD1 inhibitors | 7.452 | 7.872 | 0.0012 |
|  | CTLA-4 blockers | 6.57 | 6.863 | 0.0384 |
|  | Combination theraoy | 6.065 | 6.390 | 0.0344 |
| GBM | PD1 inhibitors | 6.179 | 6.414 | 0.4109 |
|  | CTLA-4 blockers | 6.607 | 6.724 | 0.6530 |
|  | Combination theraoy | 5.286 | 5.621 | 0.2885 |
