# Supplementary figures and images for: Gender specific eRNA TBX5-AS1 as the immunological biomarker for male patients with lung squamous cell carcinoma in pan-cancer screening
Source: PeerJ. 2021 Nov 25;9:e12536. doi: 10.7717/peerj.12536 (PMC8627656; doi:10.7717/peerj.12536)

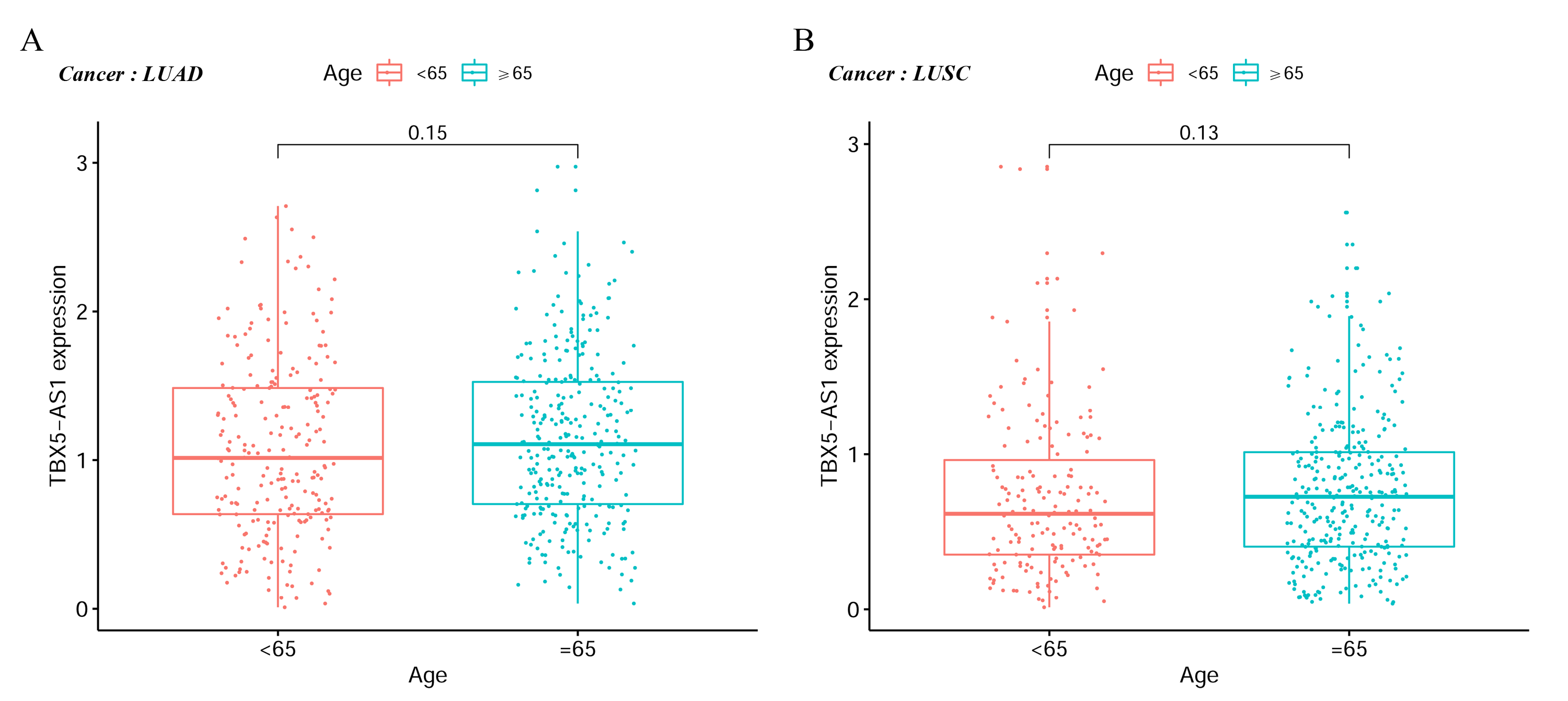

Supplement: Supplemental Information 5 [file peerj-09-12536-s005.png]

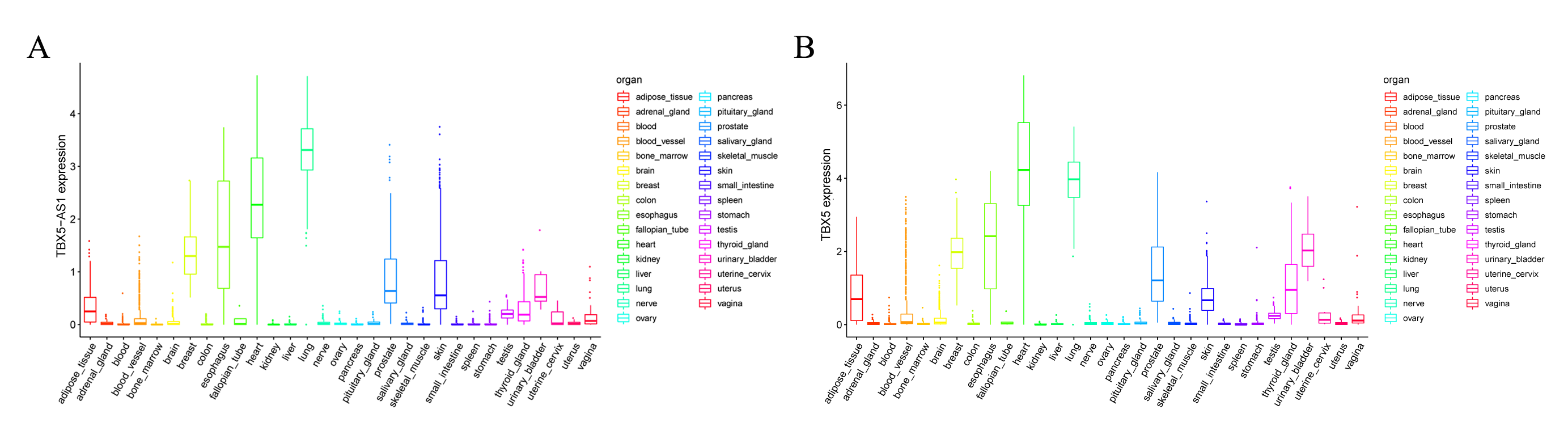

Supplement: Supplemental Information 6 [file peerj-09-12536-s006.png]

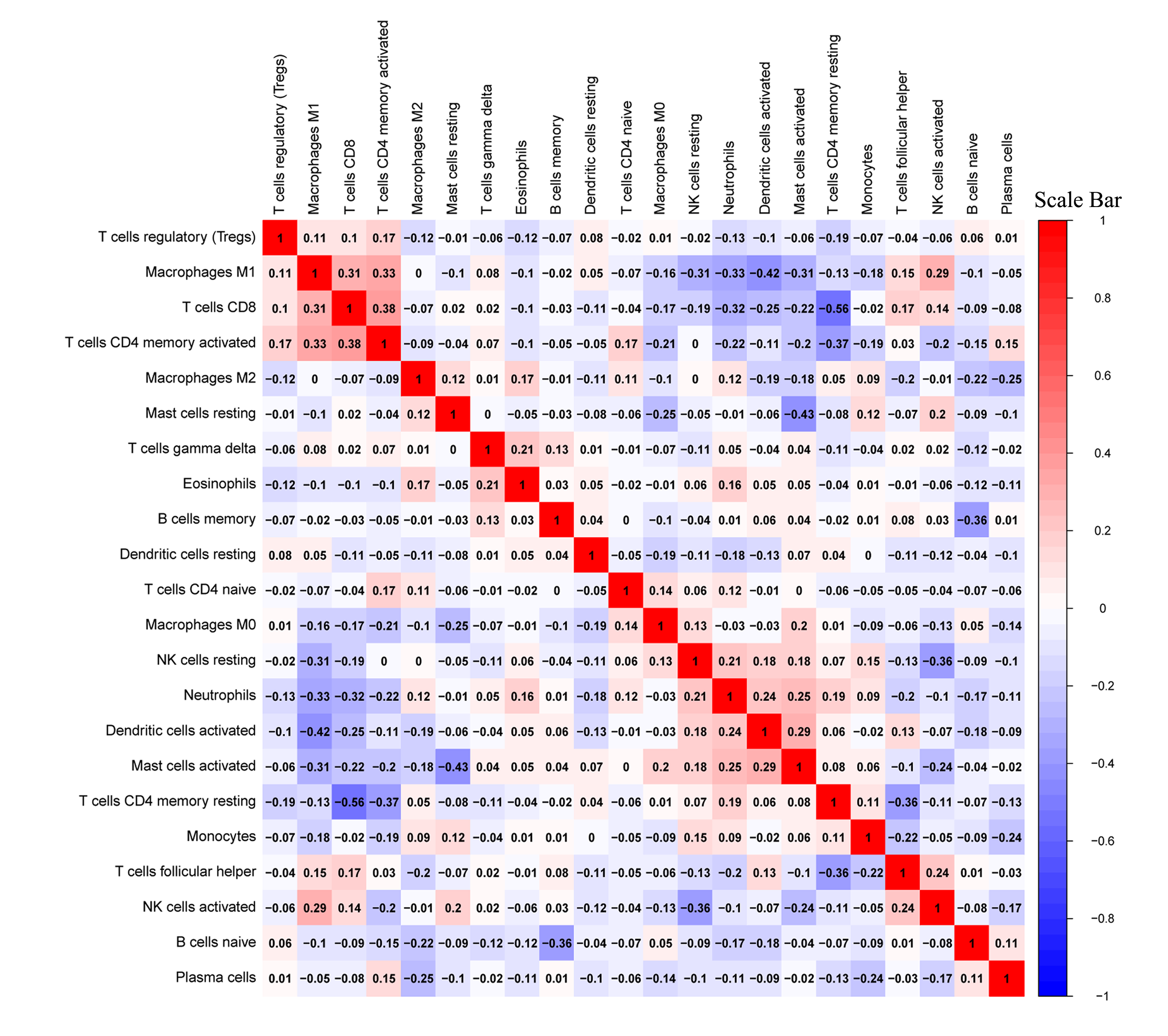

Supplement: Supplemental Information 7 [file peerj-09-12536-s007.png]
